# Functional importance of bile acid-FXR signaling in neonatal immunity and disease

**DOI:** 10.1038/s41423-025-01337-z

**Published:** 2025-08-29

**Authors:** Douglas G. Burrin, Greg Guthrie, Caitlin Vonderohe

**Affiliations:** USDA/ARS Children’s Nutrition Research Center, Section of Pediatric Gastroenterology, Hepatology and Nutrition, Department of Pediatrics, Baylor College of Medicine, Houston, TX USA

**Keywords:** Inflammation, Diagnostic markers

**Fig. 1:**
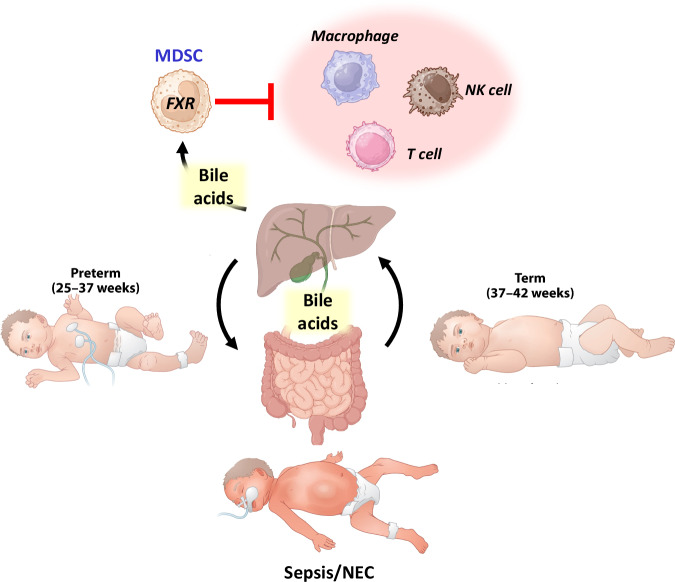
Overview of the birth transition in human infants and the role of bile acid homeostasis in FXR function in MDSCs

The neonatal period is critical for infant survival, as newborns transition from maternal umbilical oxygenation and nourishment to independent respiration and oral nutrient assimilation via the gastrointestinal tract. For preterm infants, this transition is life-threatening because of immature lung and gut function. Advances in medical care have improved preterm survival, but these infants remain highly susceptible to infection and inflammation-driven diseases, such as sepsis and necrotizing enterocolitis (NEC) [1, 2] (Fig. 1). A prevailing hypothesis attributes this vulnerability to an immature immune system, both innate and adaptive, that is unprepared for microbial colonization postbirth. However, recent studies from the Zhou laboratory highlighted a key immune cell population, myeloid-derived suppressor cells (MDSCs), which combat infection and control inflammation in neonatal inflammatory disease models [3, 4]. In this context, He et al. [5] linked bile acid signaling through the farnesoid X receptor (FXR) to enhanced MDSC function, offering protection against sepsis in mice. Using obeticholic acid (a clinically approved FXR agonist) and Fxr knockout mice, they demonstrated that FXR activation reduces inflammation and tissue injury in sepsis. Fxr deficiency impairs MDSC immunosuppressive function, whereas conditional FXR deletion in Fxrfl/flMrp8-Cre+ mice disrupts spleen-derived MDSC activity, with HIF-1 identified as a downstream FXR target. Notably, transplanting wild-type MDSCs into Fxr-deficient mice restored protection against sepsis-related inflammation and injury.

This study highlights a novel role for bile acids in neonatal immunity. Extensive literature describes the role of bile acid receptors, including FXR, in modulating immune cells such as monocytes, macrophages, CD4+ T cells (Th17, Tregs), dendritic cells, type 3 innate lymphoid cells, and NKT cells [6]. However, their role in late perinatal and neonatal immunity remains poorly understood. At term birth, hepatic bile acid synthesis increases to support dietary fat digestion from breast milk or formula, a process that is less robust in preterm infants and contributes to poor fat absorption. Our prior work revealed that, compared with preterm piglets, term neonatal piglets exhibit increased bile acid synthesis and gut‒liver FXR signaling, driving increased fibroblast growth factor 19 (FGF19) secretion [7]. The intestinal secretion of FGF19 is triggered by luminal bile acid activation of epithelial cell FXR, which serves as an important negative feedback hormone that suppresses hepatic expression of cholesterol 7-alpha-hydroxylase, the rate-limiting enzyme in bile acid synthesis. Conversely, a recent Zhou laboratory study reported a negative correlation between plasma FGF19 and gestational age in human newborns [8]. We also linked a late-gestation cortisol surge, amplified by vaginal birth, to increased FGF19 secretion, which is consistent with evidence that cortisol increases hepatic bile acid synthesis and secretion. These findings suggest that the cortisol-driven increase in bile acid production prepares neonates for extrauterine life. The physiological role of cortisol in bile acid homeostasis in preterm infants may be further complicated by the common therapeutic use of glucocorticoids for the maturation of lung function. The results of He et al. raise the following question: does immature bile acid production or FXR expression contribute to immune dysfunction in preterm infants, and does therapeutic use of glucocorticoids impact immune function via altered bile acid homeostasis?

Dysregulated bile acid homeostasis is implicated in pediatric diseases. The findings of He et al. align with the anti-inflammatory role of bile acids across immune cells, suggesting that FXR activation enhances MDSC function to dampen inflammation [6]. In addition to FXR, immune cells express receptors such as TGR5/GPBAR1, which are activated by secondary bile acids (e.g., deoxycholic acid, lithocholic acid) produced by the gut microbiota [6]. In infants, primary bile acids (chenodeoxycholic acid (CDCA) and cholic acid (CA)) predominate, and CDCA potently activates FXR. Other bile acid species, such as hyocholic acid, which are poor FXR agonists, are more abundant in preterm infants than in term infants. Thus, the bile acid profile and gut microbial colonization influence FXR function. The current He et al. [5] report is one of the first to show the efficacy of the FXR agonist obeticholic acid, given orally to neonates at a relatively high dose (20 mg/kg) without any reported apparent toxicity. However, the effect of FXR activation is not uniform, underscoring the importance of the inflammatory context and tissue-specific regulation. For example, elevated serum bile acids are associated with inflammatory diseases such as sepsis and NEC [9], yet lower bile acid levels are correlated with early-onset sepsis in infants. Further highlighting this tissue-specific complexity, Zhang et al. recently reported that FXR ablation in the intestine protects against NEC via a ferroptosis-mediated pathway involving type 3 innate lymphoid cells, suggesting context-specific roles for FXR in different inflammatory diseases [8]. Parenteral nutrition, which is common in preterm infants, disrupts bile acid homeostasis by inducing cholestasis, increasing the risk of sepsis [10]. Similarly, advanced neonatal liver diseases, such as biliary atresia, involve immune dysfunction and cholestatic bile acid accumulation, driving hepatic inflammation and fibrosis [11]. In the context of these chronic elevated bile acid diseases, MDSCs may have contrasting responses on the basis of their length of activation. In biliary atresia, a condition characterized by progressive inflammation and fibrosis of the bile ducts, MDSCs are thought to modulate the inflammatory microenvironment. In BA, excessive immune activation, particularly involving CD8+ T cells and innate immune cells such as macrophages, drives bile duct injury. MDSCs may counteract this by dampening these overactive immune responses, potentially limiting tissue damage but also contributing to immune evasion by pathogenic processes. While MDSCs can suppress acute inflammation, their chronic presence in the liver microenvironment may promote fibrosis, a hallmark of advanced liver diseases such as BA. MDSCs produce factors such as transforming growth factor-beta (TGF-β) and interleukin-10 (IL-10), which can drive fibroblast activation and extracellular matrix deposition, leading to progressive scarring. In BA, fibrotic obstruction of bile ducts is a key feature. MDSCs may exacerbate this process by creating an immunosuppressive environment that allows unchecked fibrogenesis, potentially worsening liver damage over time.

He et al.’s work highlights the therapeutic potential of FXR activation in pediatric inflammatory diseases while revealing significant knowledge gaps in bile acid and receptor function in neonatal immunity. Emerging techniques such as single-cell RNA sequencing and spatial transcriptomics enable detailed analysis of bile acid receptor signaling in specific immune cell types in neonatal blood, intestine, and liver. These tools could clarify the role of FXR and bile acids in immune responses, paving the way for targeted interventions to improve preterm infant outcomes.
